# Editorial: Environmental effect on neuroinflammation and neurodegeneration, volume II

**DOI:** 10.3389/fncel.2023.1269180

**Published:** 2023-08-11

**Authors:** Souvarish Sarkar, Srikant Rangaraju, Claudia Espinosa-Garcia, Monica Renee Langley

**Affiliations:** ^1^Department of Environmental Medicine, University of Rochester Medical Center, Rochester, NY, United States; ^2^Department of Neurology, Emory University, Atlanta, GA, United States; ^3^Department of Molecular Pharmacology and Experimental Therapeutics, Mayo Clinic, Rochester, MN, United States

**Keywords:** neuroinflammation, neurodegeneration, microglia, pesticides, epigenetics

## Introduction

Emerging epidemiological data in the last decade have indicated that environmental factors like pesticides, and metals, among others, play a critical role in driving neurodegenerative disorders like Alzheimer's disease (AD), Parkinson's disease (PD), and others. Recent research in cell culture and animal models has further shown that exposure to these neurotoxicants leads to neuroinflammation and neurodegeneration through various mechanisms. With the field of environmental toxicology gaining prominence, this set of articles in the Research Topic titled “*Environmental effects on Neuroinflammation and neurodegeneration volume II*” concentrated on the potential role of epigenetics in regulating neuroinflammation as well as the mechanism of various environmental factors contributing to neurodegeneration.

## Epigenetics and neuroinflammation and neurodegneration

Recent studies have shown that immune cells can memorize exposure to a chemical or micro-organism and then mount a more robust immune response when exposed to similar xenobiotics or chemicals. This concept is known as trained immunity. Huang et al. demonstrated both *in vitro and in vivo* that microglial cells, the brain's resident immune cells, exhibit trained immunity in response to a neurotoxic metal, manganese, that has been shown to increase the risk of PD. Furthermore, this study showed that epigenetic markers modulate this trained immune response in microglia cells in vitro and in mice in response to LPS priming and subsequent Mn exposure. H3K27ac and H3K4me3, along with H3K4me1, were all upregulated, leading to microglial cells mounting an enhanced response.

IFN-β was one of the first disease-modifying therapies approved for Multiple Sclerosis. In a clinical study, by Xavier et al. demonstrated that IFN-β treatment reduced the whole blood DNA methylation profiles of various genes that are targeted by interferons. This study suggests that epigenetic markers play a key role on MS etiology and drive neuroinflammation and neurodegeneration.

## Neurodegeneration and pesticide exposure

Meyer et al. presented novel work on the NADPH oxidase inhibitor, mitoapocynin, as a feasible countermeasure in an animal model of organophosphate (OP) toxicity. Their study specifically uses a rat model of diisopropylflurorophospate (DFP) exposure to demonstrate promising improvements in several inflammatory and oxidative stress markers in the serum of mitoapocynin-treated mice 1-week post-challenge; although NOX2 protein upregulation in response to DFP as well as reactive gliosis indicators was not attenuated in the brain tissue. Overall, the study highlights the need for follow-up dose optimization studies for promising compounds to effectively dampen environmental responses in neuroinflammatory and neurodegenerative effects in the central nervous system. Although the conceivable utility of mitoapocynin has now been shown across several rodent models of neurodegenerative and neuroinflammatory conditions, careful and sufficient dosing or perhaps alternative administration routes will need to be considered to fully overcome reactive gliosis and oxidative damage in the CNS. This is especially true for OPs, for which there are no effective medical countermeasures to mitigate chronic health effects from resulting exposures.

## Pollution and amyotrophic lateral sclerosis

Saucier et al. published a systematic review, finding almost 50 epidemiological studies evaluating alleged connections among urbanization, air pollution, and water pollution with the development of amyotrophic lateral sclerosis (ALS), also known as Lou Gehrig's disease. Importantly, this study cast a wider net to look at several exposure routes within the same systematic review as well as branched out from previous works that focused on rural settings or single contaminants of concern. Moreover, very few studies have carefully assessed the quality of individual articles included in reviews discussing environmental factors possibly linked to ALS. Although urbanization was the most well-studied, there was no clear association with ALS. In terms of air pollution as a risk factor, diesel exhaust exposure and its primary product of combustion, nitrogen dioxide, were linked to an increased risk of ALS. Water pollution also had two potential risk factors of ALS: heavy metal contamination, selenium, as well as proximity of residence to lakes prone to cyanobacterial blooms.

This Research Topic highlights how our epigenome may play a critical role in driving neuroinflammation and in the pathogenesis of neurological diseases. Innate, as well as adaptive immunity, may also play important roles in driving neuroinflammation through trained immunity. With the dawn on exposomics as well as our increase in knowledge about the effects of per- and polyfluorinated alkyl substances (PFAS), also known as “forever chemicals,” we anticipate that the interactions between environmental exposures and mechanisms of neuroinflammatory and neurodegenerative diseases will be an active area of research for years to come.

## Author contributions

SS: Writing—original draft, Writing—review and editing. SR: Writing—review and editing. CE-G: Writing—review and editing. ML: Writing—original draft, Writing—review and editing.

